# Characterisation of Gas-Chromatographic Poly(Siloxane) Stationary Phases by Theoretical Molecular Descriptors and Prediction of McReynolds Constants

**DOI:** 10.3390/ijms20092120

**Published:** 2019-04-29

**Authors:** Angelo A. D’Archivio, Andrea Giannitto

**Affiliations:** Dipartimento di Scienze Fisiche e Chimiche, Università degli Studi dell’Aquila, Via Vetoio, Coppito, 67100 L’Aquila, Italy; andrea.giannitto@gmail.com

**Keywords:** gas chromatography, poly(siloxane) stationary phases, QSRR modelling, molecular descriptors, retention prediction, McReynolds constants

## Abstract

Retention in gas–liquid chromatography is mainly governed by the extent of intermolecular interactions between the solute and the stationary phase. While molecular descriptors of computational origin are commonly used to encode the effect of the solute structure in quantitative structure–retention relationship (QSRR) approaches, characterisation of stationary phases is historically based on empirical scales, the McReynolds system of phase constants being one of the most popular. In this work, poly(siloxane) stationary phases, which occupy a dominant position in modern gas–liquid chromatography, were characterised by theoretical molecular descriptors. With this aim, the first five McReynolds constants of 29 columns were modelled by multilinear regression (MLR) coupled with genetic algorithm (GA) variable selection applied to the molecular descriptors provided by software Dragon. The generalisation ability of the established GA-MLR models, evaluated by both external prediction and repeated calibration/evaluation splitting, was better than that reported in analogous studies regarding nonpolymeric (molecular) stationary phases. Principal component analysis on the significant molecular descriptors allowed to classify the poly(siloxanes) according to their chemical composition and partitioning properties. Development of QSRR-based models combining molecular descriptors of both solutes and stationary phases, which will be applied to transfer retention data among different columns, is in progress.

## 1. Introduction

Quantitative structure–retention relationship (QSRR) method is a specialised branch of quantitative structure–activity(property) relationship (QSA(P)R) approach aimed at relating the retention of the analytes in separation chromatographic systems to their molecular structure [1,2,3]. Multilinear regression (MLR) and, less often, partial-least square or artificial neural network regression have been used to establish the relationship between the solute structure, encoded by a set of molecular descriptors, and the retention time (or a related parameter) observed in liquid or gas chromatography [1]. A QSRR model, once calibrated on a sufficiently large set of representative solutes by multivariate regression of the measured retentions against the molecular descriptors, can be applied to deduce the chromatographic behaviour of unseen compounds solely from their chemical structure, although prediction is restricted to the same column/mobile phase pair and separation mode used in calibration. With the aim of extending retention prediction to a useful domain of separation conditions, which can be of great help in the optimisation of the chromatographic methods usually based on time-consuming empirical approaches, QSRR-based comprehensive models combining solute molecular descriptors and descriptors of the mobile phase [4,5,6] or the column [7,8] have been recently proposed.

Retention in gas chromatography (GC) is mainly governed by the extent of the intermolecular interactions between the solutes and the stationary phase, since the gaseous mobile phase is not involved in the partition mechanism under the typical experimental conditions of analytical separations [9]. In this regard, the ability of the stationary phase of being involved in inductive, dispersive, orientation, lone-pair electron and H-bonding interactions determines the column polarity, while selectivity is related with its capacity to participate in specific intermolecular interactions. In linear free energy relationships (LFERs) and LFER-based QSRRs [9,10], empirical or semiempirical molecular descriptors have been conceived to quantify the abilities of the solutes to take part in the postulated intermolecular interactions with the chromatographic phases. In spite of their conceptual and historical importance, the LFER molecular descriptors are not readily available for most solutes of current analytical interest. On the other hand, structural properties provided by quantum chemistry or thousands of molecular descriptors determined by a large spectrum of other computational methods are nowadays available for encoding the effect of molecular structure in QSAR modelling. As regards QSRRs, several classes of theoretical molecular descriptors, often with no simple physical identity, have been employed in the last decades to model the retention behaviour of many different chemical classes on specific GC separation systems [1,11,12,13,14,15].

While theoretical characterisation of solutes is a consolidated practice in QSRR modelling, description and classification of the GC stationary phases is historically based on empirical approaches enduring until recently [9]. One of the most popular empirical scales is the McReynolds system of phase constants [16], which is based on the difference in the retention index values for prototypical solutes on the stationary phase to be characterised and on squalane, taken as a nonpolar reference phase. Apart from classification of columns, in terms of polarity/selectivity, empirical descriptors of the stationary phase, including the McReynolds constants, were previously used in combination with theoretical molecular descriptors of solutes with the aim of transferring retention data among different columns [17,18,19]. In this context, the possibility of describing by computational molecular descriptors not only the effect of the solute structure on the retention but also the main partitioning properties of GC stationary phases is an attractive objective.

Poly(siloxane) stationary phases (Table 1 and Table 2) occupy a dominant position in modern liquid–gas chromatography (LGC), because of excellent thermal and chemical stability coupled with high solute diffusivity [20]. Moreover, the polarity and selectivity of LGC poly(siloxane) columns can be widely tuned by varying the kind and content of the functional groups incorporated into the structure [21]. In this paper, a QSSR method focusing on the column rather than on the solute was developed with the aim of characterising the poly(siloxane) stationary phases by means of theoretical molecular descriptors. To identify among the large number of structural properties provided by popular software Dragon [22] a small set able to represent the partitioning ability of poly(siloxanes), the McReynolds constants were considered as QSRR responses. We established a specific QSRR for each of the first five McReynolds constants, X, Y, Z, U and S, based on the prototype solutes benzene, butanol, 2-pentanone, nitropropane and pyridine, respectively, selected to characterise the principal intermolecular interactions responsible for retention. The ability of the McReynolds solutes to represent individual intermolecular interactions has been sometimes criticised [21]. Nevertheless, X, Y, Z, U and S seem the adequate responses of a QSRR model focused on the stationary phase chemical structure, regardless of their capability of providing a quantitative measure of the stationary phase selectivity, since each quantity refers to a specific solute and was determined with a standardised experimental protocol in which any other source of variability, related for instance with the column geometry or the elution conditions, was removed. The McReynolds constants of molecular (nonpolymeric) stationary phases were previously modelled by QSRR using quantum chemical descriptors [23,24], while the molecular descriptors here employed were obtained by less sophisticated and faster computational methods. The polymeric structure of poly(siloxane) stationary phases, by contrast, makes their theoretical characterisation not so obvious as in the case of the molecular stationary phases previously investigated. The QSRR models were generated by MLR coupled with genetic algorithm (GA) variable selection. Principal component analysis (PCA) was applied to the set of the significant molecular descriptors to support the physical interpretation of the final QSRR models and attempt classification of poly(siloxane) columns.

## 2. Results and Discussion

### 2.1. QSRR Dataset

The dataset investigated in this work consists of 29 poly(siloxane) stationary phases belonging to poly(methylphenylsiloxane), poly(methyltrifluoropropylsiloxane) and poly(cyanoalkylmethylphenylsiloxane) subgroups displayed in Table 1 and Table 2 together with the first five McReynolds constants X, Y, Z, U and S, taken from scientific [25] or commercial literature [26]. The code of a GLC commercial column is associated to each stationary phase, but it must be noted that many equivalent poly(siloxane)-based columns can be provided by different manufacturers [20].

Poly(dimethylsiloxane) (column OV-1) is a nonpolar and low-selectivity phase that can be regarded as the basic structure of the stationary phases here investigated. Substitution of methyl groups with phenyl, trifluoropropyl and cyanoalkyl groups in variable concentration permits extending the selectivity of the poly(siloxanes) over a wide range [25], which makes them the most versatile in GLC analytical separations.

**Figure 1:**
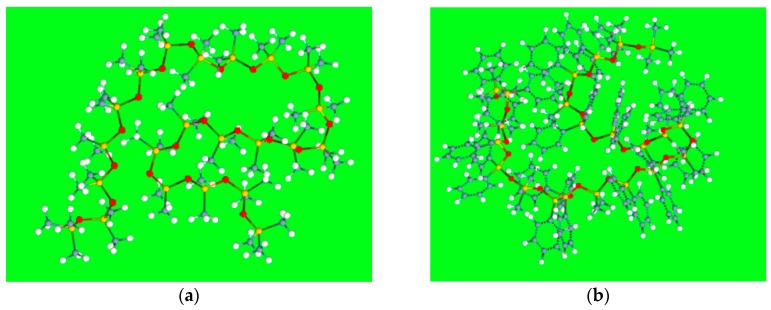
Geometrical models of OV-1 (**a**) and Rtx-65 (**b**) stationary phases. Grey, white, yellow and red colours identify C, H, Si and O atoms, respectively.

In previous analogous investigations [23,24] aimed at modelling the McReynolds constants of nonpolymeric stationary phases (esters of dicarboxylic acids, for instance) the molecular descriptors were determined using a standard procedure, consisting of a preliminary geometry optimisation followed by the computation of the structural properties. Polysiloxane stationary phases, by contrast, are polymers with a high molecular weight (generally in the range of 10^3^ to 10^6^) [25], and therefore a simplified geometrical model must be generated. In this work, each stationary phase was represented by an oligomer formed by 20 siloxane units ending with trimethylsiloxy groups. This choice represents a good compromise between the needs of an acceptable computation time and adequate representation of the bulk properties of the stationary phase. In variously-substituted poly(siloxanes), the different comonomers were uniformly positioned within the polysiloxane backbone. In few cases (columns OV-61, FS-328, FS-169 and NSKT-33), the requisite of having an integer number of each comonomer resulted in a slight deviation of the geometrical composition in the geometrical model compared to the nominal one. Figure 1 displays the optimised molecular models of poly(dimethylsiloxane) (column OV-1) and 65% diphenyl-35% dimethyl poly(siloxane) (column Rtx-65). Regardless of the chemical composition, the polysiloxane backbone in the optimised structures is coiled to favour the attractive intrachain interactions. In spite of the much lower polymerisation degree and the absence of interchain interactions, the optimised geometrical models should provide a reliable representation of the reciprocal position of the siloxane substituents within the real stationary phases which governs the retention of solutes and column selectivity. The optimised structures were processed by computer package Dragon 6 which provides 4885 molecular descriptors belonging to various classes [27]. However, to avoid including redundant variables in the QSRR dataset, the descriptors with little variance were removed, and only one descriptor was retained among groups of highly correlated ones (*r* > 0.85). After this preliminary variable selection, 177 molecular descriptors were identified and stored for further analysis.

### 2.2. QSRR Modelling of McReynolds Constants by GA-MLR

A specific QSRR model was established for each of the five McReynolds constants by multilinear regression (MLR) coupled with genetic algorithm (GA) variable selection [28,29]. Before developing the QSRR models, we designed an external prediction set by selecting five columns (OV-7, OV-25, SILAR 7CP, XE-60 and FS-328) covering as far as possible the structural variability of the 29 poly(siloxanes) in terms of qualitative and quantitative composition. A preliminary GA-MLR exploration was carried out to identify the optimal complexity of the QSRRs. We observed that including six descriptors into the various QSRR models gave satisfactory results, while incorporation of a seventh descriptor produced only a negligible improvement of Q^2^_loo-cv_. Therefore, the maximum number of descriptors to be selected by GA was set to six. Some hundreds of GA-MLR runs with different starting chromosome populations were performed for each of the five responses and the descriptors selected at least one time were collected together in a same data set that was subjected to a final GA-MLR analysis. The models with the highest Q^2^_loo-cv_ values for each of the five different responses were finally chosen. These are presented in Table 3, while the selected molecular descriptors are listed in Table 4.

**Figure 2:**
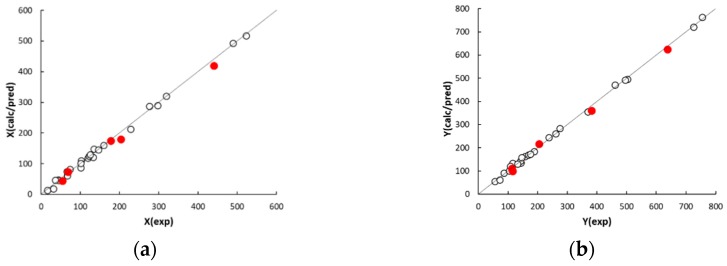

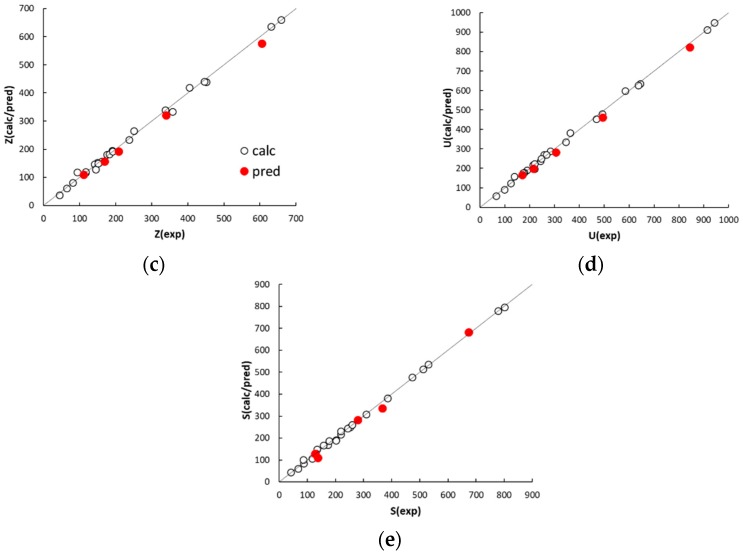
Agreement between experimental McReynolds constants X (**a**), Y (**b**), Z (**c**), U (**d**) and S (**e**), and calculated (white circles) or predicted (red circles) QSRR responses.

**Figure 3:**
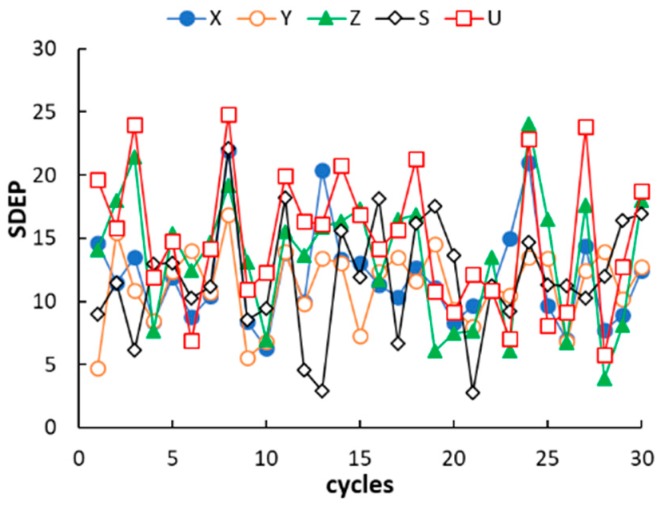
Trend of standard deviation of the error in prediction (SDEP) in repeated prediction/calibration random partitions of Monte Carlo validation.

The agreement plots of the computed or predicted McReynolds constants and the experimental values (displayed in Figure 2) reveal a distribution of both calibration and prediction data samples close to the ideal line. The observed determination coefficients of calibration and external prediction, R^2^ and Q^2^ (displayed in Table 3), fall within 0.9964 to 0.9986 and 0.9867 to 0.9940, respectively, suggesting a good descriptive and predictive performance of the five QSRR models. The values of standard deviation of the error in calibration (SDEC) and prediction (SDEP) are within 9 to 12 and 15 to 21, respectively. In this regard, it must be noted that phases with McReynolds constants differing by within ±10 units generally exhibit a same separation performance [26]. The individual residuals associated to the calibration columns (displayed in Table A2, Appendix A) are randomly distributed around zero and only in a limited number of cases fall outside the ±10 range, which suggests a very good fitting. As expected, the model residuals in prediction are higher than those observed in calibration but worsening of the model performance is anyway acceptable. It must be noted that most of the residuals in prediction are positive. This trend, however, was not observed in leave-one-out cross validation, which leads to exclude the effect of systematic errors in QSRR prediction. It follows that the partitioning properties of poly(siloxane) stationary phases can be predicted with acceptable accuracy by QSRR modelling. Apart from using the preselected external set, the generalisation ability of the QSRR models was further evaluated for different partitions of the columns between the calibration and prediction sets. Following a repeated (or Monte Carlo) validation scheme, 30 random partitions were generated with an average of 20% of columns in each prediction set. The mean SDEP value and the associated standard deviation observed for each response is given in Table 3, while the SDEP trend over the 30 repetitions is displayed in Figure 3. The number of external columns in repetitions ranged from two to nine; it follows that structural variability may be not adequately represented by the calibration set when a relatively high number of columns belonging to a same subgroup is transferred in the prediction set. Nevertheless, the mean and individual SDEP values in repetitions confirmed the good generalisation ability of the QSRR models observed in external prediction. The QSRRs previously developed to model the McReynolds constants of nonpolymeric (molecular) stationary phases using semiempirical quantum chemical descriptors can be considered for comparison. In a first study, regarding 25 stationary phases (phthalates, adipates, sebacates, phosphates, citrates and nitrils) [24], all the 10 McReynolds constants were simultaneously modelled by partial least-square regression and the observed Q^2^ values related to various external sets consisting of six columns ranged between 0.9736 and 0.9834. In a successive investigation [23], the McReynolds constants of 36 nonpolymeric stationary phases were modelled by MLR, seven of the investigated columns being selected for external prediction. The SDEP values associated to the first five McReynolds were found to fall between 27 and 50. Therefore, generalisation ability of the QSRR models for the poly(siloxane) stationary phases here developed is better than that reported in literature for nonpolymeric columns, despite poly(siloxanes) are more complex structures and less sophisticated molecular descriptors were used to describe their partitioning properties.

### 2.3. Interpretation of the QSRR Models

The molecular descriptors selected in the QSRR modelling of the five McReynolds constants are collected in Table 2. Table 3 displays the regression coefficient b of each significant molecular descriptor, while its relative importance in defining the QSRR response is quantified by the standardised b value (b’). The values of the selected molecular descriptors associated to the 29 poly(siloxane) stationary phases are listed in Table A1 (Appendix A).

**Figure 4:**
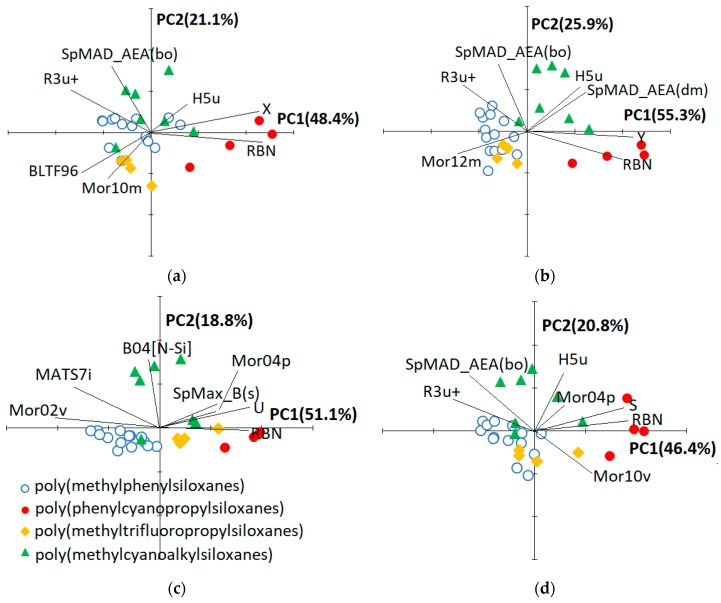
Projection of columns and variables of QSRRs established for X (**a**), Y (**b**), U (**c**) and S (**d**) in the plane of the first two principal components.

To facilitate the physical interpretation of the QSRR models, PCA was performed on the autoscaled QSRR variables (descriptors and response) and both scores (columns) and loadings (variables) were plotted in the plane of the first two principal components (Figure 4). Based on the three plots (not shown) reporting the variance explained by each PC, the third principal component seems to be also significant. Nevertheless, to have a simple graphical representation of the PCA results, we considered only the first two PCs, that together account for a percentage of total variance ranging between 67% and 81% (Figure 4). The biplots associated to the QSRR model for Z and U are almost identical according to the fact that the same set of molecular descriptors was selected, and the two responses are highly correlated, therefore, PCA results referring to the first case were not reported in Figure 4. PCA also offers a graphical tool to rank the chromatographic phases, this approach being already used to classify the columns based on various kind of empirical descriptors [9,21,30,31]. It must be noted that the plots displayed in Figure 4 do not change appreciably if the experimental response is removed from the variable set subjected to PCA.

Figure 4 reveals that the 29 poly(siloxane) stationary phases are generally grouped according to their chemical composition, but the reciprocal position of the columns in the PC1−PC2 plane is also dependent on the QSRR response and, therefore, on the set of molecular descriptors entering the model. To explain this finding it must be reminded that the first five McReynolds constants X, Y, Z, U and S are associated to predefined compounds able to establish specific interactions [9]: benzene (weak proton acceptor and π−π interactions), butanol (proton donor and proton acceptor interactions), 2−pentanone (proton acceptor interactions), nitropropane (dipole interactions) and pyridine (strong proton acceptor interactions), respectively. Regarding the stationary phases [21,25], progressive introduction of phenyl groups in poly(methylphenylsiloxanes) influences the column selectivity because of both a strong dispersion interaction and a high polarisability of the phenyl groups compared to a methyl group. Poly(trifluoropropylmethylsiloxanes) are moderately polar; their selectivity is based on the pronounced acceptor character of the 3,3,3−trifluoropropyl group that can interact with free electron pairs. Cyanoalkyl-containing polysiloxanes are the most polar stationary phases. The cyano group, attached to the siloxane backbone via two or three CH_2_ groups, is dipolar and strongly electron attracting. It is therefore able to display dipole−dipole, dipole-induced dipole and charge-transfer interactions. Moreover, the unshared electron pair on the nitrile nitrogen can promote hydrogen-bonding interactions with H-donor solutes. It follows that differentiation of the columns in the subspace of the significant PCs extracted from the QSRR descriptors should reflect the ability of the related McReynolds solute to interact with the various poly(siloxane) stationary phases.

The number of rotatable bonds (RBN) is the most influent molecular descriptor regardless of the QSRR response, according to the higher b’ value of this variable in all the generated models (Table 3). The contribution of the various siloxane substituents to RBN follows the order methyl < phenyl < cyanoethyl < trifluoropropyl = cyanopropyl that closely reflects the polarity order for these groups. B04[N−Si] (presence/absence of N−Si at topological distance 4) is the second most important descriptor in QSRR models for Z and U (Table 3); its value is 1 for cyanoethyl-containing poly(siloxanes) (NPS−100, NSKI−25, NSKT−33 and XE−60) and 0 for all the other phases (Table A1). The second most important descriptor in QSRR models for X, Y and S is SpMAD_AEA(bo) (spectral mean absolute deviation from augmented edge adjacency matrix weighted by bond order) that partially duplicates structural information of B04[N−Si], according to the moderate correlation between these two quantities (*r* = 0.79, Table A3); the other descriptors entering the various models seem to describe minor structural effects, according to their relatively low b’ values.

About the ability of the selected molecular descriptors for the classification of the poly(siloxane) stationary phases, Figure 4 reveals that poly(phenylcyanopropylsiloxanes)(SILAR 5CP, SILAR 7CP, SILAR 9 CP and SILAR 10 CP) are well separated from the others along PC1, regardless of the QSRR response which is almost colinear with PC1 itself. These four stationary phases are also differentiated to each other along PC1 according to the increasing ratio of cyanopropyl to phenyl substituents when the QSRR response is X (Figure 4a) or Y (Figure 4b). This finding can be explained by the ability of the related prototype solutes benzene and butanol to strongly interact with cyano groups by means of dipole-induced dipole and H bonding interactions, respectively. Poly(methylcyanoalkilsiloxane) stationary phases, on the other hand, are always separated from the others along a direction approximately parallel to PC2 and the most external columns are those containing cyanoethyl groups (NPS−100, NSKI−25, NSKT−33 and XE−60), while poly(methylcyanopropylsiloxanes) are closer to the origin of the PC1−PC2 graph. It follows that poly(methylcyanoethylsiloxanes) and poly(methylcyanopropylsiloxanes) can be discriminated by the selected molecular descriptors (B04[N−Si] or SpMAD_AEA(bo) in particular, as previously discussed). The reciprocal position of poly(methylphenylsiloxane), poly(methyltrifluoropropylsiloxane) and poly(methylcyanopropylsiloxane) columns along PC1, that, as previously discussed, is almost colinear with the QSRR response, is moderately influenced by the kind of solute. These three subgroups are poorly separated when the prototype solute is benzene (X, Figure 4a) or pyridine (S, Figure 4d), which may be attributed to the ability of these two aromatic solutes to establish both π−π and dipole-induced dipole interactions. By contrast, poly(methylcyanoalkylsiloxanes) retain butanol (Y, Figure 4b) more than both poly(methyphenylsiloxanes) and poly(methyltrifluoropropylsiloxanes), which indicates a relevant role of H bonding interactions between the alcoholic group of this prototype solute and the cyano groups of the stationary phase. The difference between the behaviour of poly(methylcyanoalkylsiloxanes) and poly(methyltrifluoropropylsiloxanes) is almost negligible when U (Figure 4c) or Z is the QSRR response, but the two prototype solutes 2-pentanone and nitropropane are less retained by the poly(methylphenylsiloxane) stationary phases. This pattern can be explained by the dominant role of the dipole−dipole interactions between each of the two solutes and the stationary phases. In summary, the theoretical molecular descriptors entering the QSRR models, apart from allowing the accurate prediction of the McReynolds constants, can be used to classify the stationary phases by means of PCA.

## 3. Materials and Methods

### 3.1. Structure Generation and Molecular Descriptor Calculation

Starting geometries of the poly(siloxanes) were drawn by means of the MacroModel 7.1 molecular modelling program package [32] assuming standard bond lengths and angles. The global energy minimum of each molecule was searched using the MM2 force field. To avoid staking in local minima, geometry optimisation was repeated on several starting conformers randomly generated. Software Dragon 6 [22] was used to compute the molecular descriptors from the optimised geometrical models of the stationary phases. The version utilised in this work provides 4885 descriptors classified as zero- (0D), one- (1D), two- (2D) and three-dimensional (3D) descriptors depending on the fact they are computed from the chemical formula, the substructure list representation, the molecular graph or the geometrical representation of the molecule, respectively. After removal of constant and highly correlated variables (*r* > 0.85), only 177 molecular descriptors were retained for further analyses.

### 3.2. Development of QSSR Models

A specific QSRR model was established for each of the five McReynolds constants, X, Y, Z, U and S, by multilinear regression (MLR) combined with genetic algorithm (GA) selection to identify a small subset of significant variables within the 177 molecular descriptors provided by Dragon. MLR is the most convenient multivariate tool for QSRR modelling, because of simple statistical bases and easy interpretation of the resulting models: the retention time or a related parameter is expressed as a linear combination of molecular descriptors and the related coefficients are determined by ordinary least squares regression [33]. However, when many molecular descriptors are available, MLR must be combined with a suitable variable selection method to identify a small subset of significant and uncorrelated descriptors. In the GA−MLR method, regression models are represented by chromosomes, namely binary vectors in which the value 1 or 0 of each position (or gene) encodes the presence or absence, respectively, of a descriptor in the model. A starting random population of chromosomes, alias regression models, evolves for several generations by application of cross-over and mutation rules inspired by principles of natural selection and genetics until an optimal or near optimal model is identified. In the crossover process two mating chromosomes exchange their genetic material according to the “uniform crossover technique”, in which for each gene a random number determines if it will undergo crossover or not. Mutation is caused by a random change of the value of a gene based on very low selected probability (here 0.1%). The chance for each chromosome of passing to the next generation is quantified by a “fitness function”, which, in regression problems, is the model predictive performance, expressed here by the determination coefficient in leave-one-out cross−validation (Q^2^_loo−cv_). To avoid the loss of highly predictive models, a predefined number of the best chromosomes (elitism, here fixed to 1%) are passed unchanged to the next generation. In this work, the initial population consists of 100 chromosomes and evolution is carried out until no further improvement of Q^2^_loo−cv_ of the best model occurs after five cycles.

### 3.3. Model Validation

The descriptive performance of each MLR model was evaluated by usual statistical parameters [33]: the coefficient of determination and the standard deviation of the error in calibration (R^2^ and SDEC, respectively). The predictive ability of the QSRR models was quantified by the coefficient of determination and the standard deviation of the error evaluated on a predesigned external set (Q^2^ and SDEP, respectively), Q^2^ being computed according to Todeschini et al. [34]. The predictive performance of the established QSRRs was further evaluated by Monte Carlo or repeated test set validation [33]. In this method, a large number of training and test sets are randomly generated with a preselected probability of assignment and the SDEP is computed on the total number of predictions.

### 3.4. Principal Component Analysis

Principal component analysis (PCA) was used to help interpretation of the QSRR models and attempt unsupervised classification of the stationary phases based on their molecular structure. PCA [35] allows to represent multivariate information in a reduced subspace of principal components (PCs), namely orthogonal directions, the first describing the largest variance, the second describing the second−largest variance, and so on. PCs are obtained by a proper orthogonal rotation around the centroid of the data after variable autoscaling, which produces the diagonalisation of the correlation matrix. In the rotated space the new variables (the PCs) are not correlated and are ordered according to their variance (eigenvalue). The coordinates of the objects in the space of PCs are called “scores,” and the orthogonal rotation matrix with the direction cosines is called matrix of “loadings”. Projecting the objects scores and variables loadings into the space of few significant PCs allows revealing patterns in the original data matrix with minimal loss of information. All the statistical analyses were performed using the program package V−PARVUS 2010 [36].

## 4. Conclusions

In the present study, poly(siloxane) stationary phases, the most widely used in liquid−gas chromatography and historically classified by empirical polarity/selectivity scales, were characterised using theoretical molecular descriptors. In spite of the polymeric nature of these stationary phases, the selected molecular descriptors allowed for prediction of the McReynolds constants with acceptable accuracy and are useful to classify the columns according to their partitioning properties. Further work is in progress to evaluate the performance of QSRR−based predictive models combining molecular descriptors of both stationary phases and solutes.

## Figures and Tables

**Table 1:** Molecular structure of poly(methylphenylsiloxane) stationary phases and related McReynolds constants.

POLY(METHYLPHENYLSILOXANES) 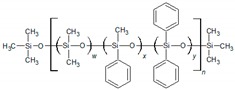								
Column	Composition (%)			McReynolds Constants				
	w	x	y	X	Y	Z	U	S
OV-1	100	0	0	16	55	44	65	42
SE-52	95	5	0	32	72	65	98	67
OV-3	80	20	0	44	86	81	124	88
OV-7	60	40	0	69	113	111	171	128
DC-550	50	50	0	74	116	117	178	135
OV-11	30	70	0	102	142	145	219	178
OV-17	0	100	0	119	158	162	243	202
SP-392	0	90	10	133	169	176	258	219
OV-22	0	70	30	160	188	191	283	253
OV-25	0	50	50	178	204	208	305	280
Rtx-20	80	0	20	67	116	117	174	131
OV-61	67(70) ^a^	0	33(30) ^a^	101	143	142	213	174
Rtx-35	65	0	35	101	146	151	219	202
Rtx-65	35	0	65	125	175	183	268	220

^a^ nominal composition and in brackets that of the geometric model.

**Table 2:** Molecular structure of poly(methyltrifluoropropylsiloxane) and poly(cyanoalkylmethylphenylsiloxane) stationary phases, and related McReynolds constants.

**POLY(METHYLTRIFLUOROPROPYLSILOXANES)** 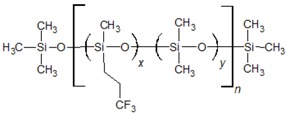															
**Column**		**Composition (%)**					**McReynolds Constants**								
		**x**			**y**		**X**		**Y**		**Z**		**U**	**S**	
OV-210		100			0		146		238		358		468	310	
SKIFT-50X		50			50		66		132		192		247	158	
FS-328		31(30) ^a^			69(70) ^a^		55		116		169		215	137	
FS-169		23(25) ^a^			77(75) ^a^		46		104		149		189	118	
**POLY(CYANOALKYLMETHYLPHENYLSILOXANES)** 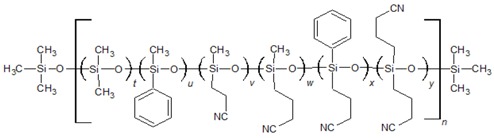															
**Column**	**Composition (%)**									**McReynolds Constants**					
	**t**		**u**	**v**		**w**	**x**	**y**		**X**		**Y**	**Z**	**U**	**S**
SILAR 5CP	0		0	0		0	100	0		319		495	446	637	530
SILAR 7CP	0		0	0		0	50	50		440		638	605	844	673
SILAR 9CP	0		0	0		0	20	80		489		725	631	913	778
SILAR 10CP	0		0	0		0	0	100		523		755	659	942	801
OV-105	90		0	0		10	0	0		36		108	93	139	86
OV-225	0		50	0		50	0	0		228		369	338	492	386
*p*-NSKT-100	0		0	0		100	0	0		276		461	405	584	473
NPS-100	0		0	100		0	0	0		297		502	451	644	512
NSKI-25	75		0	25		0	0	0		122		261	237	345	244
NSKT-33	67(65) ^a^		0	33(35) ^a^		0	0	0		135		275	251	363	259
XE-60	50		0	50		0	0	0		204		381	340	493	367

^a^ nominal composition and in brackets that of the geometric model.

**Table 3:** Significant molecular descriptors of the established quantitative structure–retention relationship (QSRR) models, multilinear regression (MLR) coefficients (b) and related standardised values (b’); descriptive and predictive performance evaluated on the external prediction set and by Monte Carlo (MC) validation.

Response	Descriptors	MLR Coefficients		Calibration		Prediction		MC Validation
		b_i_(±_Sb_)	b_i_’	R^2^	SDEC	Q^2^	SDEP	SDEP (±SD)
X+	intercept	−1996(±203)	−	0.9964	9	0.9944	15	12 (±1)
	RBN	3.83(±0.13)	0.93					
	Mor10m	−4.6(±0.9)	−0.08					
	R3u+	−4327(±1725)	−0.06					
	BLTF96	−1.7(±0.8)	−0.05					
	SpMAD_AEA(bo)	1132(±136)	0.19					
	H5u	22(±6)	0.07					
Y	intercept	−3861(±234)	−	0.9984	9	0.9867	15	11 (±2)
	RBN	199(±41)	0.86					
	SpMAD_AEA(dm)	5.18(±0.11)	0.14					
	Mor12m	−4.4(±0.9)	−0.07					
	R3u+	−10,190(±1998)	−0.08					
	SpMAD_AEA(bo)	2190(±175)	0.24					
	H5u	22(±7)	0.04					
Z	intercept	−89(±23)	−	0.9966	12	0.9900	18	14 (±5)
	RBN	4.7(±0.1)	0.92					
	SpMax_B(s)	18(±2)	0.13					
	MATS7i	1635(±270)	0.14					
	Mor02v	−0.7(±0.2)	−0.07					
	Mor04p	2.1(±0.5)	0.08					
	B04[N-Si]	87(±9)	0.19					
U	intercept	−87(±25)	−	0.9981	12	0.9920	21	15 (±4)
	RBN	6.94(±0.12)	0.94					
	SpMax_B(s)	18(±3)	0.09					
	MATS7i	2449(±283)	0.17					
	Mor02v	−0.9(±0.2)	−0.07					
	Mor04p	2.9(±0.5)	0.08					
	B04[N-Si]	133(±10)	0.21					
S	intercept	−4119(±173)	−	0.9986	9	0.9914	19	12 (±5)
	RBN	6.16(±0.09)	0.98					
	SpMAD_AEA(bo)	2543(±111)	0.29					
	Mor10v	−6.6(±1.4)	−0.05					
	Mor04p	2.0(±0.3)	0.06					
	H5u	19(±5)	0.05					
	R3u+	−14,847(±1723)	−0.13					

**Table 4:** Meaning and class of the molecular descriptors of the QSRR models.

Name	Description	Kind
RBN	number of rotatable bonds	Constitutional indices
B04[N−Si]	presence/absence of N–Si at topological distance 4	2D Atom Pairs
MATS7i	Moran autocorrelation of lag 7 weighted by ionisation potential	2D autocorrelations
SpMAD_AEA(dm)	spectral mean absolute deviation from augmented edge adjacency mat. weighted by dipole moment	Edge adjacency indices
SpMAD_AEA(bo)	spectral mean absolute deviation from augmented edge adjacency mat. weighted by bond order	
SpMax_B(s)	leading eigenvalue from Burden matrix weighted by I−State	2D matrix−based descriptors
H5u	H autocorrelation of lag 5/unweighted	GETAWAY descriptors
R3u+	R maximal autocorrelation of lag 3/unweighted	
Mor02v	signal 02/weighted by volume	3D−MoRSE descriptors
Mor10v	signal 10/weighted by volume	
Mor10m	signal 10/weighted by mass	
Mor12m	signal 12/weighted by mass	
Mor04p	signal 04/weighted by polarisability	
BLTF96	Verhaar Fish base−line toxicity from MLOGP (mmol/l)	Molecular properties

## Abbreviations

GA	Genetic algorithm
GC	Gas−chromatography
GLC	Gas−liquid chromatography
QSRR	Quantitative structure−retention relationship
QSA(P)R	Quantitative structure−activity(property) relationship
MLR	Multilinear regression
PCA	Principal component analysis
SDEC	Standard deviation of the error in calibration
SDEP	Standard deviation of the error in prediction

## Appendix A

**Table A1:** Values of the significant molecular descriptors associated to the 29 poly(siloxanes) stationary phases.

Column	RBN	B04[N-Si]	MATS7i	SpMAD_AEA(dm)	SpMAD_AEA(bo)	SpMax_B(s)	H5u	R3u+	Mor02v	Mor10v	Mor10m	Mor12m	Mor04p	BLTF96
OV-1	42	0	−0.053	1.545	1.545	4.809	7.117	0.008	42.211	−0.858	4.642	15.951	−21.884	2.37
SE-52	43	0	−0.053	1.542	1.556	4.814	7.083	0.01	35.619	−0.634	5.137	16.165	−15.074	1.76
OV-3	46	0	−0.042	1.536	1.573	4.837	6.703	0.012	38.626	−1.81	2.55	14.571	−19.435	−0.19
OV-7	50	0	−0.028	1.531	1.579	4.863	6.476	0.011	44.266	−2.377	2.13	18.579	−24.66	−1.77
DC-550	52	0	−0.025	1.528	1.579	4.875	6.469	0.011	72.16	−2.163	2.082	15.778	−19.586	−2.51
OV-61	54	0	−0.032	1.527	1.578	4.894	6.072	0.01	29.779	−2.072	1.723	17.356	−17.733	−3.22
OV-11	56	0	−0.021	1.525	1.576	4.9	6.27	0.009	73.701	−3.18	0.103	20.357	−25.407	−3.9
OV-17	62	0	−0.023	1.521	1.569	4.933	6.129	0.008	58.168	0.039	2.456	20.795	−28.92	−5.8
SP-392	64	0	−0.03	1.52	1.566	4.945	6.243	0.008	58.085	0.802	3.711	20.538	−16.688	−6.39
OV-22	68	0	−0.042	1.519	1.561	4.969	6.571	0.008	21.562	−2.996	−0.789	21.118	−21.29	−7.53
OV-25	72	0	−0.058	1.517	1.556	4.993	6.639	0.007	29.796	−2.231	−0.147	18.624	−17.043	−8.61
Rtx-20	50	0	−0.046	1.53	1.579	4.867	6.324	0.012	37.429	−0.593	3.002	17.031	−8.291	−1.77
Rtx-35	56	0	−0.034	1.525	1.576	4.903	6.167	0.009	35.2	−1.401	1.404	16.429	−17.796	−3.9
Rtx-65	68	0	−0.05	1.519	1.561	4.974	5.679	0.007	23.124	−0.382	2.383	21.372	−20.894	−7.53
OV-105	48	0	−0.049	1.63	1.558	6.817	6.945	0.009	36.061	−0.939	3.664	15.231	−19.314	2.92
NPS-100	82	1	−0.023	1.901	1.626	6.855	6.956	0.009	22.53	−2.242	−0.291	16.092	−9.307	−7.67
OV-225	82	0	−0.039	1.671	1.564	6.834	7.048	0.008	17.747	−2.293	−0.61	9.448	−4.936	0.7
SILAR 5CP	122	0	−0.079	1.669	1.543	6.857	6.763	0.006	2.658	2.693	4.434	18.009	−20.383	1.28
SILAR 7CP	142	0	−0.073	1.724	1.54	6.878	6.93	0.006	−7.61	1.753	1.886	13.495	−9.339	−4.96
SILAR 9CP	154	0	−0.068	1.754	1.537	6.89	7.259	0.006	−6.983	−2.676	−1.881	7.273	−11.534	−8.51
SILAR 10C	162	0	−0.065	1.779	1.536	6.898	7.205	0.005	−16.129	0.531	1.127	9.927	−22.813	−11.77
OV-210	82	0	−0.062	1.52	1.549	8.588	6.527	0.007	5.555	2.691	10.992	20.14	−8.637	−5.44
SKIFT-50X	62	0	−0.073	1.59	1.548	8.548	6.14	0.008	36.349	0.481	5.378	20.805	−12.166	−1.88
FS-328	54	0	−0.07	1.604	1.547	8.532	6.264	0.009	34.562	−1.088	4.363	17.002	−13.577	−0.3
FS-169	52	0	−0.069	1.603	1.547	8.528	6.319	0.008	15.486	−1.436	3.316	16.587	−18.31	0.12
NSKI-25	52	1	−0.038	1.726	1.595	6.823	6.785	0.007	33.814	−1.908	2.002	14.529	−23.779	−2.01
NSKT-33	56	1	−0.031	1.772	1.606	6.828	7.357	0.011	51.199	−1.89	2.213	15.363	−18.17	−2.77
XE-60	62	1	−0.026	1.822	1.617	6.834	7.326	0.01	33.34	−0.468	4.235	14.863	−15.124	−3.9
p-NSKT-100	103	0	−0.041	1.792	1.556	6.855	6.864	0.007	22.687	−0.329	1.201	10.956	−10.858	−5.52

**Table A2:** Calculated and predicted residuals of the QSRR models.

Column	QSRR Response				
	X	Y	Z	U	S
OV−1	3	0	7	7	−2
SE−52	13	11	5	6	7
OV−3	−3	−5	−1	0	4
FS−169	0	2	−3	−1	11
DC−550	−8	−17	1	0	−14
OV−61	14	7	−4	−5	5
OV−11	−8	−1	16	22	−9
OV−17	0	−5	5	5	12
SP−392	12	−1	−5	−13	3
OV−22	0	4	−5	−6	3
NSKI−25	−2	0	3	9	−1
Rtx−20	5	12	−2	−3	0
Rtx−35	−1	−12	2	−3	14
Rtx−65	−5	2	0	−1	−12
OV−105	−10	−12	−25	−20	−16
NPS−100	7	6	11	9	−1
OV−225	15	13	−1	13	5
SILAR 5CP	−2	2	6	10	−5
NSKT−33	−13	−8	−14	−19	−1
SILAR 9CP	−4	4	−5	1	−2
SILAR 10C	6	−7	−2	−8	5
OV−210	0	−6	24	14	2
SKIFT−50X	−9	1	0	−3	−10
p−NSKT−100	−11	−9	−14	−15	−4
OV−7 ^a^	−4	2	2	5	0
OV−25 ^a^	3	−12	16	22	−2
SILAR 7CP ^a^	20	14	30	21	−9
XE−60 ^a^	24	21	20	32	31
FS−328 ^a^	11	17	12	17	26

^a^ external set.

## Appendix B

**Table A3:** Correlation matrix of the molecular descriptors representing the poly(siloxane) stationary phases.

	RBN	B04[N-Si]	MATS7i	SpMAD_AEA(dm)	SpMAD_AEA(bo)	SpMax_B(s)	H5u	R3u+	Mor02v	Mor10v	Mor10m	Mor12m	Mor04p	BLTF96
RBN	1.0000	−0.1153	−0.4768	0.5296	−0.4353	0.3243	0.3771	−0.7280	−0.7776	0.3623	−0.2683	−0.5350	0.2520	−0.5450
B04[N-Si]		1.0000	0.3848	0.6562	0.7943	0.2100	0.4322	0.1693	0.0907	−0.1816	−0.0750	−0.1283	0.0280	−0.0752
MATS7i			1.0000	−0.0462	0.7886	−0.5553	−0.0859	0.5546	0.6889	−0.5045	−0.2966	0.1581	−0.2872	−0.0897
SpMAD_AEA(dm)				1.0000	0.2934	0.5110	0.6866	−0.2749	−0.4441	0.0416	−0.2604	−0.6418	0.3264	−0.2119
SpMAD_AEA(bo)					1.0000	−0.2115	0.0933	0.5848	0.4669	−0.4036	−0.1746	0.1056	−0.0471	−0.0244
SpMax_B(s)						1.0000	0.2171	−0.3834	−0.4893	0.3587	0.3428	−0.2461	0.4437	0.0663
H5u							1.0000	−0.1077	−0.3377	−0.0359	−0.1340	−0.7048	0.2288	0.0616
R3u+								1.0000	0.6582	−0.4222	0.0723	0.1889	−0.0307	0.4387
Mor02v									1.0000	−0.3809	0.0332	0.4743	−0.4028	0.2970
Mor10v										1.0000	0.6901	0.1487	0.1902	0.0083
Mor10m											1.0000	0.3833	0.0989	0.3848
Mor12m												1.0000	−0.3682	0.0184
Mor04p													1.0000	0.0109
BLTF96														1.0000
